# Acute effects of lower limb wearable resistance on horizontal deceleration and change of direction biomechanics

**DOI:** 10.1371/journal.pone.0308536

**Published:** 2024-09-09

**Authors:** Nicolas M. Philipp, Quincy R. Johnson, Dimitrije Cabarkapa, Andrew C. Fry

**Affiliations:** Jayhawk Athletic Performance Laboratory, Wu Tsai Human Performance Alliance–University of Kansas, University of Kansas, Lawrence, KS, United States of America; Università degli Studi di Milano: Universita degli Studi di Milano, ITALY

## Abstract

This study aimed to investigate the acute effects of lower limb wearable resistance on maximal horizontal deceleration biomechanics, across two different assessments. Twenty recreationally trained team sport athletes performed acceleration to deceleration assessments (ADA), and 5-0-5 change of direction (COD) tests across three load conditions (unloaded, 2% of BW, 4% of body weight (BW)), with load attached to the anterior and posterior thighs and shanks. Linear mixed effect models with participant ID as the random effect, and load condition as the fixed effect were used to study load-specific biomechanical differences in deceleration mechanics across both tests. Primary study findings indicate that for the ADA, in the 4% BW condition, participants exhibited significantly greater degrees of Avg Approach Momentum, as well as significant reductions in deceleration phase center of mass (COM) drop, and Avg Brake Step ground contact deceleration (GCD) in both the 2% BW, and 4% BW condition, compared to the unloaded condition. In the 5-0-5 tests, participants experienced significant reductions in Avg Approach Velocity, Avg deceleration (DEC), and Stopping Time in the 4% BW condition compared to the unloaded condition. Similar to the ADA test, participants also experienced significant reductions in Avg Brake Step GCD in both the 2% BW and 4% BW conditions, and significant increases in Avg Approach Momentum in the 4% BW condition, compared to the unloaded condition. Therefore, findings suggest that based on the test, and metric of interest, the addition of lower limb wearable resistance led to acute differences in maximal horizontal deceleration biomechanics. However, future investigations are warranted to further explore if the use of lower limb wearable resistance could present as an effective training tool in enhancing athlete’s horizontal deceleration and change of direction performance.

## Introduction

In addition to straight line running, the majority of court and field-based sports require athletes to perform high-intensity locomotive tasks in all three planes of motion (e.g., cutting, jumping, lateral movement) [1]. For instance, Taylor et al. suggested that studies in soccer reported the most frequent cutting (up to 800 per game), while studies in basketball reported the highest frequency of lateral movement (up to 450 per game) [1]. In many cases, these cuts and change of direction maneuvers are preceded by rapid reductions in the athlete’s horizontal velocity and whole-body momentum. Gaining more attention recently, investigations into athletes’ maximal horizontal deceleration mechanics have documented that compared to sprint accelerations, the braking steps during decelerative tasks possess a unique ground reaction force profile characterized by high-impact peak forces and loading rates when compared to sprint accelerations [2]. Peak vertical ground reaction forces during high-intensity decelerations have been shown to reach magnitudes of ~5.9 times body weight, which is roughly 2.7 times greater than the peak ground reaction forces experienced during maximal horizontal acceleration [2, 3]. From a kinematic standpoint, when compared to horizontal sprint accelerations, during the deceleration phase, especially at ground contact, athletes strike the ground with the heel first instead of the ball of the foot, with a dorsiflexed ankle, fairly extended knee, slightly flexed hip, and an erect or slightly posteriorly leaning torso [4]. Horizontal deceleration ability has been suggested to be underpinned by modifiable factors related to a deceleration skill component, as well as neuromuscular deceleration qualities (e.g., strength) [5]. With potential implications on athlete health and performance, deceleration ability has rightfully achieved increased levels of interest within the sport science community. However, given the recency of this trend, and the fact that deceleration research is still in its’ infancy stages, there is a lack of evidence-backed training modalities that have the potential to improve athlete’s deceleration abilities. While not in as much breadth and depth as linear sprint performance, change of direction ability and associated training modalities have received more considerable attention within the scientific literature [6–11]. For instance, research has shown that technique modification, as well as plyometric and resistance training modalities may improve change of direction performance [6, 7, 11, 12].

Linear sprint performance has long been acknowledged as a cornerstone of athletic development within most strength and conditioning departments. Therefore, an extensive body of literature has devoted efforts towards investigating methods to assess and improve physical qualities related to sprint performance [13–16]. Under the sprint performance enhancement umbrella, one rather novel training modality that has been gaining increased popularity over recent years is the implementation of lower limb wearable resistance. Compared to other forms of resisted running, such as sled pulling and pushing, this modality involves the use of light additional loads (e.g., 1–5% of body mass), attached to different parts of the body, such as the ankle, shank, thigh, trunk, as well as the arms. While speculative in nature, the primary interest in this training modality stems from the suspected lack of transfer from gym-based resistance training to actual improvements in sprint performance [17]. Most research on lower body wearable resistance for sprint performance has studied the acute effects of this training stimulus on biomechanics of sprint running. For instance, according to Simperingham and Cronin (2014), treadmill sprinting with an additional load of 5% of body mass attached to the lower body resulted in a significant reduction in step frequency, reduced velocity after the initial 10 meters, as well as increased ground contact times and increased ground reaction forces [18]. Further, Simperingham et al. (2016) proposed that using an additional load of 3% and 5% of body mass attached around the subjects’ calves during 20-meter sprints led to no significant decreases in split times over the initial 10 meters of the sprint [19]. However, the 5% loading strategy led to slower 20-meter times, compared to both the unloaded and 3% loading strategies. According to the authors, the added lower body loading was well tolerated during the leg pumping or “piston-like” action of the initial acceleration phase [19]. Authors concluded that a moderate additional load (i.e., 3% of body mass) attached to the calves provides a loading stimulus that serves to increase horizontal force output during the acceleration phase of sprinting [19]. Lastly, Macadam et al. (2017) who used wearable resistance equivalent to 3% of body mass around the quadriceps, tibialis anterior, hamstrings, and gastrocnemius, suggested that similar to the previous study, split times over the initial 10 meters were not affected by the loading strategy [20]. However, 10–20-meter split times were significantly affected as seen by a slower split time (-2 to 3%) and a significantly lower theoretical maximum velocity achieved (-5 to 6%) [20].

Feser et al. (2021) recently studied the effects of using a rather light (1% of body mass) wearable resistance loading strategy over nine weeks on sprint performance within a group of high school football players [15]. Post-intervention, no statistically significant differences were found between groups (intervention vs. control). However, both groups saw improvements in velocity measures following the training intervention, suggesting that both training modalities had a positive impact on sprint performance. Further, Bustos et al. (2020) investigated the effects of warming up with lower-limb wearable resistance over the course of eight weeks on physical performance measures within a group of national-level U20 soccer players [21]. Primary findings from the study were that the wearable resistance group experienced significantly larger improvements in 10- and 20-meter sprint times for the entire pre-, to post-training cycle, compared with the control group, with authors suggesting that sprint performance improvements stem from an increased rotational workload at the hip and knees, enough to stimulate improvements in horizontal performance capabilities, manifested as better sprint times.

It seems that while novel and exploratory, the use of lower limb wearable resistance may present itself as a promising tool with regards to maintaining or improving linear sprint performance. However, the effects of wearable resistance on other locomotive tasks (e.g., change of direction, deceleration) have been largely unexplored. To the best of the authors’ knowledge, only a few studies have tried to investigate the effects of wearable resistance on change of direction or deceleration mechanics/performance [22–24]. However, in one study, the additional load of 5% of the subjects’ body weight was attached to the torso rather than to the lower limbs [22]. Another study investigated the effects of shank vs. thigh wearable resistance on total and split times during a change of direction task consisting of 90-degree and 45-degree cuts [23]. This study concluded that lower limb wearable resistance with different loads had an acute effect on change of direction performance, and that distal placement (shank vs. thigh) with similar body mass load had a larger effect upon performance [23]. While novel and interesting, the aforementioned study merely quantified change of direction performance through completion times, and therefore failed to take into consideration more detailed biomechanical parameters related to the task. Maximal horizontal deceleration ability is likely an underpinning quality of change of direction performance, however, little is known about training modalities that could potentially influence or improve this physical construct in an isolated manner. Furthermore, a greater body of evidence exists investigating the later decelerative steps during change of direction maneuvers (i.e., penultimate and final foot contact) [25, 26], while less is known about the biomechanics of the earlier decelerative steps during maximal deceleration tasks. To the authors best knowledge, no previous studies have tried to quantify the effects of lower limb wearable resistance on an isolated measure of horizontal deceleration ability, as well as change of direction performance.

Therefore, the aim of this study was to investigate the effects of lower limb wearable resistance around the thigh and shank on the biomechanics of maximal horizontal deceleration and change of direction performance across two different assessments. We hypothesized that in the wearable resistance condition, subjects may express altered movement characteristics, such as reduced magnitudes of acceleration and deceleration, as well as potentially altered movement strategies related to center of mass displacement, lower limb joint angles, as well as ground contact times for instance, and that this could present as a potential training tool for sport science practitioners to use. Gaining further insights into the underlying biomechanical features of horizontal deceleration and change of direction tasks may allow sport science practitioners to further individualize or enhance their approach towards training athletes for such demands.

## Methods

### Experimental approach to the problem

This study was approved by the University of Kansas’s institutional review board. IRB ID: STUDY00149611. A total of 20 healthy recreationally active athletes with at least four years of experience participating in a multi-directional sport (e.g., basketball, soccer, tennis) volunteered to participate in the study. In the following order, participant’s written consent was obtained, followed by the collection of subject anthropometrics (e.g., height and weight). Following this, further anthropometric measurements and inertial measurement unit (IMU) (Xsens MVN Awinda, Enschede, the Netherlands) placement were done according to manufacturer guidelines [27–29]. From there the IMU-based system was calibrated according to manufacturer guidelines [27]. Prior to the start of data collection, all subjects performed a dynamic warm up that was led by a Certified Strength and Conditioning Specialist. The dynamic warm up consisted of general exercises such as forward and backward jogging, skips, bound, and lateral shuffles, as well as practice repetitions in both the acceleration to deceleration assessment (ADA) and the 5-0-5 change of direction test to familiarize participants with the tasks. More specifically, under the supervision of the principal investigator, each subject was given three practice repetitions for each assessment prior to the start of data collection. Upon the start of data collection, subjects were asked to perform three repetitions in the ADA as well as four repetitions (two turning off each side) in the 5-0-5 change of direction test across three load conditions (no wearable resistance, 2% BW wearable resistance, 4% BW wearable resistance). The order in which load conditions were completed was randomized for each subject.

### Subjects

A total of 20 recreationally trained, college-aged individuals (n = 15 male, age = 21.3 ± 1.9 years, height = 1.81 ± 0.07; body mass = 80.6 ± 9.6 kg; n = 5 female, age = 20.1 ± 2.8 years, height = 1.70 ± 0.02 m, weight = 63.8 ± 2.3 kg) with at least four years of recent, organized playing experience in a multidirectional sport (e.g., basketball, soccer). All participants provided their written consent prior to the start of the study, as approved by the university’s institutional review board. Participants were actively recruited between March 20^th^, 2023, and August 15^th^, 2023.

### Wearable resistance loading

**Fig 1:**
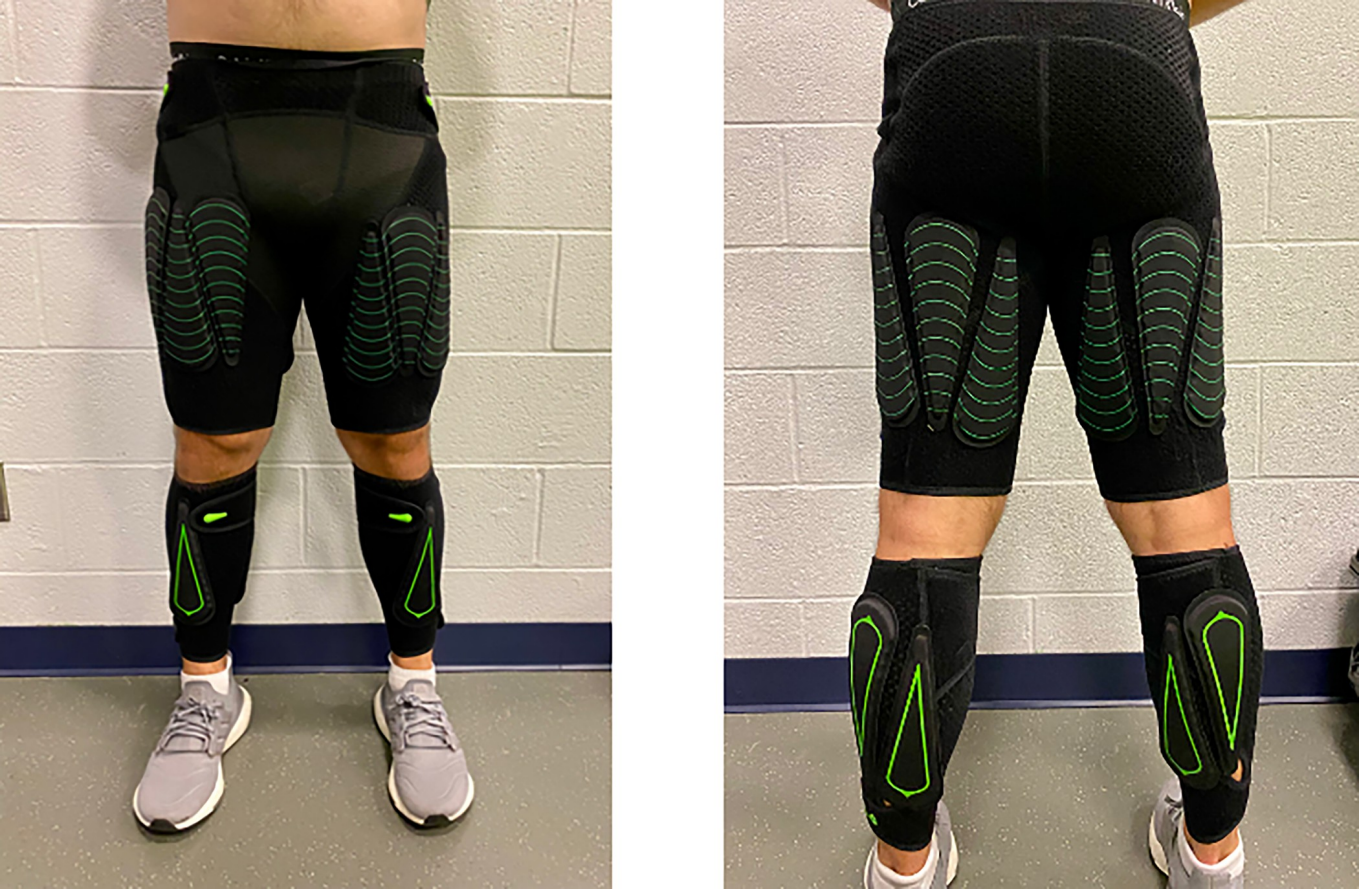
Illustration of the wearable resistance attachment, with two-thirds of the load attached to the thighs, and one-third of the load attached to the shanks.

Procedures for wearable resistance loading during the two deceleration/change of direction tasks were adapted from Busch et al. [30]. In a randomized order, participants completed three repetitions in the ADA test, as well as four repetitions (two to each side) in the 5-0-5 change of direction tests, with either no additional load, two percent of body weight (2% BW) in additional load, or four percent of additional load in body weight (4% BW). Similar to previous research, the participants wore compression shorts and calf sleeves (Lila ®Exogen™ exoskeleton suit, Sportboleh Sdh Bhd, Kuala Lumpur, Malaysia), allowing for attachment of loads from 100g to 200g. The load distribution was defined as two-thirds on the thigh and one-third on the shank [30, 31]. Therefore, loads were positioned in the middle of the frontal plane and equally distributed. If no equal distribution was possible, heavier parts were placed on the anterior part at the thigh and on the posterior part at the shank. The weight belly was placed proximal to the knee if an un- equal distribution occurred [30]. Fig 1 visually displays this distribution of wearable resistance attachments around the lower limbs.

### Acceleration to deceleration assessment (ADA)

Procedures for the execution of this maximal horizontal deceleration performance assessment were adapted from previous research [32, 33]. Subjects started in a two-point, staggered stance prior to the commencement of the test. Following a verbal signal from the principal investigator, subjects maximally accelerated over 10 meters. Timing gates (Brower Timing Systems, Draper, UT, USA) were placed at the 10-meter marker, which made a distinct sound once athletes crossed them. This sound was used as an auditory signal for participants to initiate the deceleration phase. Therefore, once crossing the 10-meteres, and hearing the sound, participants were instructed to maximally decelerate to a stop, followed by a backpedal back to the 10-meter marker. Subjects performed a total of three trials, with three minutes of passive rest in-between each trial. If athletes were visually observed to slow down prior to the 10-meter mark, or significantly after it, the trial was repeated following three minutes of passive rest.

### 5-0-5 change of direction test

Procedures for the 5-0-5 test were adapted from previous research [34], and similarly to the ADA test, started with participants in a two-point, staggered stance. Participants accelerated maximally over 10 meters, with a 180-degree turn performed at the 15-meter marker, which was marked with cones and tape. Participants crossed a single timing gate at the 10-meter marker and were instructed to sprint maximally through the same timing gate again, following the 180-degree turn and reacceleration. A researcher visually observed that participants made contact with the 15-meter marker. Participants performed two trials turning with their right leg, and two trials turning with their left leg, for each loading condition.

### Biomechanical analysis of deceleration and change of direction performance

**Fig 2:**
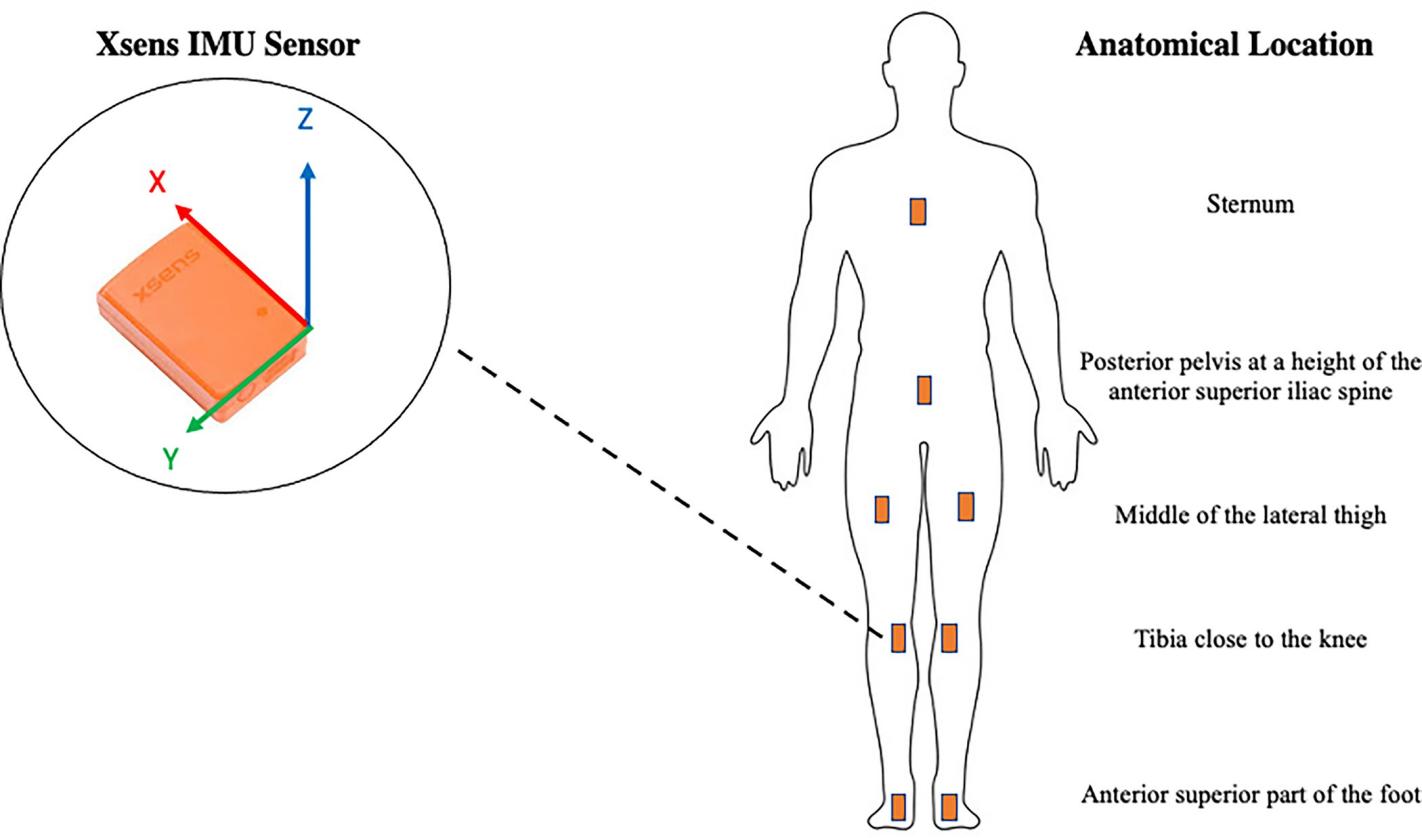
Illustration of the suit configuration “Lower body with sternum”, displaying individual IMU placement on the body.

To capture the biomechanics of horizontal deceleration and change of direction performance, researchers utilized an IMU-based motion capture system (Xsens, MVN Awinda, Netherlands). In recent research, this technology has displayed excellent agreement with a Vicon optoelectronic motion system, for all joint kinematics in the sagittal plane of movement [28]. Further, more closely aligned with the aims and procedures of this study, other research has documented validity for the proposed IMU system in assessing temporal-spatial parameters during the ADA test regardless of the preceding effort, and hip and knee kinematics following low intensity running [24]. The system consisted of motion trackers (miniature inertial measurement units) containing three-dimensional linear accelerometers, 3D magnetometers, 3D rate gyroscopes, and a barometer. In line with manufacturer guidelines, these IMU-units were placed at strategic locations on the body (secured by straps), to measure the motions of each body segment of interest. More specifically, for this study, within the Xsens MVN software (MVN Record 2023) researchers chose the suit configuration “Lower Body with Sternum”, which required units to be placed around the anterior superior part of the foot, the tibia close to the knee, the middle of the lateral thigh, the posterior pelvis at a height of the anterior superior iliac spine, as well as the sternum. This configuration is visualized in Fig 2. The individual IMU units sampled at a rate of 100 Hz, consisting of an accelerometer detecting movement, a gyroscope to measure rotation, and a magnetometer to measure orientation, allowing a single sensor in each respective location to perceive movement in multiple dimensions, which can be tracked on a cartesian coordinate system (x-, y-, and z-axes). Prior to attaching the IMU units to the subjects, researchers input subject-specific body measurements such as height and limb/segment lengths. To calibrate, the system considers the location of each IMU unit on individual segments relative to one another and combined with the subject-specific body measurements forms a biomechanical model. Raw IMU-derived data were uploaded to the Athlete Analytics software platform (Athlete Analytics, Atlanta, GA, USA) where metrics of interest were calculated based on proprietary algorithms. Metrics of interest for each assessment may be found in Table 1. For the sake of this analysis, the start of the deceleration phase was identified as the point in time where the greatest change in acceleration of the center of mass in the opposite direction (i.e., negative acceleration) occurred. This time point was further solidified, based on a proprietary algorithm, identifying the first brake step as a step exhibiting a clear increase in ground contact acceleration compared to the acceleration phase. Ground contact accelerations were derived from the accelerometers attached to the lower limbs of the participants, while their foot is in contact with the ground (x, y, and z directions combined), and were converted from meters per second to gravitational acceleration (g’s), by dividing values by 9.81. Average horizontal braking force was calculated in line with previous suggestions, using fundamental laws of dynamics in the horizontal direction (i.e., Newton’s second law of motion) [32, 35]. All joint angle-specific values were reported as relative angles.

**Table 1:** Metric names and definitions for respective assessments.

Metric (Unit)	Test	Definition | Calculation
Avg Approach Velocity (m/s)	ADA | 5-0-5	Average horizontal approach velocity of the center of mass
Avg Approach Momentum (kg*m/s)	ADA | 5-0-5	Average horizontal approach momentum of the center of mass
DEC Phase Avg. Deceleration (m/s^2^)	ADA | 5-0-5	Average horizontal deceleration of the center of mass
DEC Phase Avg. Horizontal Braking Force (N)	ADA | 5-0-5	Horizontal antero-posterior ground reaction force applied to the body COM
Stopping Time (s)	ADA | 5-0-5	Time from the start of the deceleration phase to the end of the deceleration phase
Stopping Distance (m)	ADA | 5-0-5	Distance from the start of the deceleration phase to the end of the deceleration phase
COM Drop During DEC (cm)	ADA | 5-0-5	Downward movement of the center of mass during the deceleration phase
Avg Brake Step Ground Contact DEC (g)	ADA | 5-0-5	Average ground contact deceleration during all steps of the deceleration phase
Avg Brake Step Ground Contact Time (s)	ADA | 5-0-5	Average foot ground contact time during all steps of the deceleration phase
Avg Brake Step Hip Flexion at GC (deg)	ADA | 5-0-5	Average brake step hip flexion during the deceleration phase
Avg Brake Step Knee Flexion at GC (deg)	ADA | 5-0-5	Average brake step knee flexion during the deceleration phase
Avg Brake Step Position Relative to COM (cm)	ADA | 5-0-5	Foot position relative to the center of mass for all brake steps in the deceleration phase
Change of Direction Time (s)	5-0-5	Time from the first brake step until 5 meters following 180-degree turn
Plant Leg Hip Flexion Peak (deg)	5-0-5	Maximal hip flexion of the plant leg during the 180-degree turn
Plant Leg Knee Flexion Peak (deg)	5-0-5	Maximal knee flexion of the plant leg during the 180-degree turn

*Note: DEC = Deceleration

ACC = Acceleration

COM = Center of mass

GC = Ground contact

Avg. = Average

### Statistical analyses

Researchers downloaded metrics of interest for the two assessments from the Athlete Analytics software platform (Athlete Analytics, Atlanta, GA, USA), and entered data into a excel spreadsheet, prior to importing the excel file to RStudio (Version 1.4.1106), where further data treatment and statistical analyses were performed. All data were explored for homoscedasticity and normal distribution of residuals through Q-Q plots and residuals histograms. Instead of averaging trials performed in each load condition, all trial data were used in a random intercept linear mixed effects model with restricted maximum likelihood. Compared to a traditional analysis of variance, the multilevel approach allows researchers to account for individual variability among subjects. This may be critical in our pool of subjects, given that it contains a less homogeneous group of individuals (e.g., different genders and different sporting backgrounds) In the model, respective metrics of interest from the ADA and 5-0-5 tests were entered as the dependent variable, with load condition (0% of BW vs. 2% of BW vs. 4% of BW) entered as the fixed effect, and participant ID as the random effect. In case of a significant univariate effect for load condition, further Bonferroni-corrected post-hoc comparisons were performed between the fixed factors. For all metrics of interest, within-session reliability was assessed through CV%’s for the unloaded condition. Given previously established limitations with calculating CVs through traditional methods, we used a CV calculation based on the mean square error terms of logarithmically transformed data [36, 37]. Lastly, percent differences for all significant metrics, gathered from the estimated marginal means output were compared to the CV%’s from the unloaded condition, to investigate if change occurred, greater than the metrics’ natural intra-day variability. Statistical inferences were made using an alpha level of p ≤ 0.05.

## Results

Findings from the linear mixed effect model analyses suggested several significant fixed effects across loading conditions for both assessments, while accounting for random effects (between-participant variability). Firstly, within the ADA test, significant effects for loading condition were found for Avg Approach Momentum (F = 7.55, p < 0.001), with participants generating a significantly greater approach momenta within the 4% BW condition, compared to the unloaded condition, and the 2% BW condition. Further, a significant effect for loading condition were found in the ADA test for DEC COM Drop (F = 5.93, p = 0.003), with participants experiencing a significantly shallower COM drop during the DEC phase, in the 4% BW, and 2% BW condition, compared to the unloaded condition. Similarly, significant effects for loading condition were found for Avg Brake Step GCD (F = 35.8, p < 0.00), with participants experiencing significantly smaller magnitudes of GCD within the 4% BW, and 2% BW condition, compared to the unloaded condition. Further, the 4% BW condition also suggested significantly lower Avg Brake Step GCD’s, compared to the 2% BW condition.

**Fig 3:**
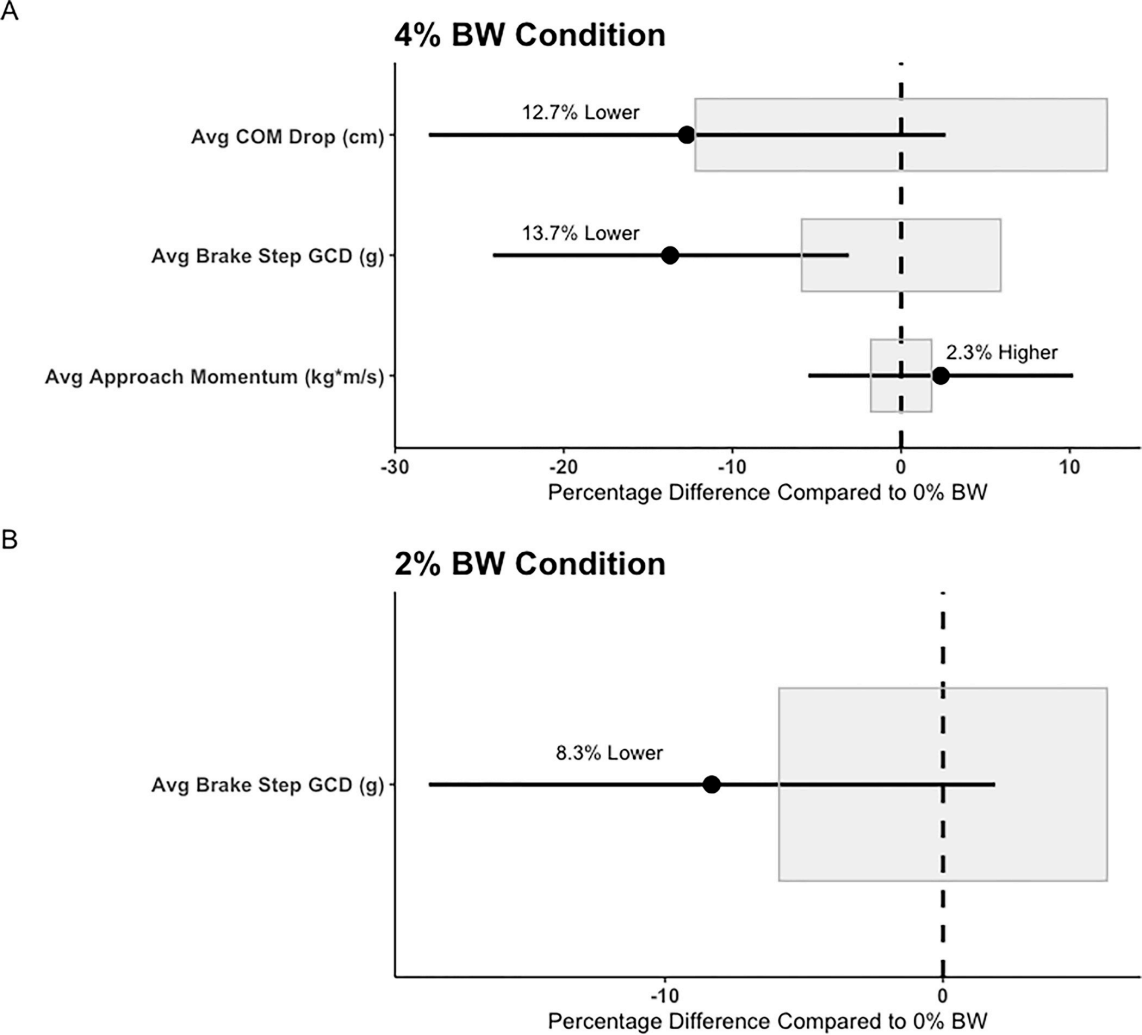
ADA mean percent difference plots for the 4% BW condition (Panel A), and the 2% BW condition (Panel B), when compared to the 0% BW condition. Points represent the mean percent difference, while the error-bar represents the 90% confidence interval. The intra-day CV% is shown for each metric as the grey area. Only significant metrics are highlighted. *Note: “GCD” = Ground contact deceleration.

**Fig 4:**
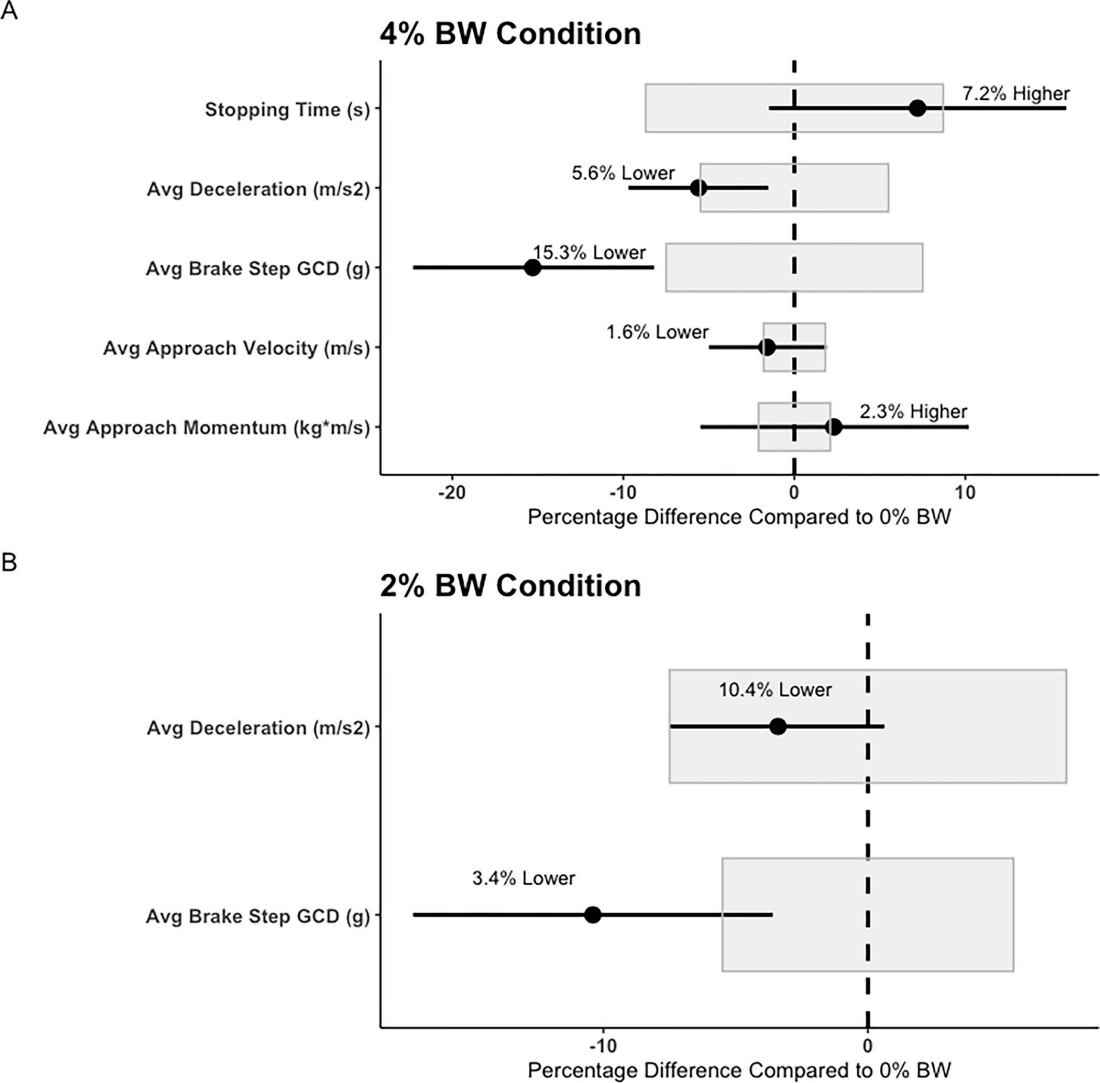
505 percent difference plots for the 4% BW condition (Panel A), and the 2% BW condition (Panel B), when compared to the 0% BW condition. Points represent the mean percent difference, while the error-bar represents the 90% confidence interval. The intra-day CV% is shown for each metric as the grey area. Only significant metrics are highlighted. *Note: “GCD” = Ground contact deceleration.

Within the 5-0-5 test, significant effects for loading condition were found for Avg Approach Velocity (F = 4.32, p = 0.014), with participants experiencing significantly lower approach velocities in the 4% BW condition, compared to the unloaded condition. Similar, significant effects for loading condition were found for Avg Approach Momentum (F = 8.80, p < 0.001). However, in this case, participants exhibited significantly larger approach momenta in the 4% BW condition, compared to the unloaded condition. During the DEC phase of the 5-0-5 test, Avg DEC showed a significant effect for loading condition (F = 8.64, p < 0.001), with participants displaying significantly lower degrees of deceleration during both the 2% BW, and 4% BW condition, compared to the unloaded condition. Interestingly, a significant effect for loading condition was also found for Stopping time (F = 3.82, p = 0.023), with participants displaying a significantly shorter Stopping time in the 4% BW condition, compared to the unloaded condition. Similar to the ADA test, within the 5-0-5 tests, a significant effect for loading condition was found for Avg Brake Step GCD (F = 27.6, p < 0.001), with participants experiencing significantly lower magnitudes of GCD during the 2% BW, and 4% BW condition, compared to the unloaded condition. Lastly, a significant univariate effect for loading condition was found for Plant Leg Hip Flexion Peak at GC (F = 3.24, p = 0.041). However, follow-up pairwise Bonferroni corrected comparisons revealed no further statistically significant differences. Lastly, out of the twelve significant condition-specific comparisons, nine suggested percentage differences greater than the intra-day CV% established in the unloaded condition. This would suggest change past the natural within-day variability of the metric. Fixed effects parameter estimates, and descriptive statistics may be found in Tables 2 and 3, respectively. Figs 3 and 4 represent the mean and 90% confidence intervals for the percentage differences between the statistically significant metrics for the 2% BW and 4% BW conditions, when compared to the 0% BW condition, as well as the intra-day CV values for respective metrics.

**Table 2:** Fixed effects parameter estimates for all metrics of interest across both assessments and all load conditions [Estimate (95% CI)].

Metric	Test	F	2% BW–Unloaded	4% BW—Unloaded
			Estimate (95% CI)	
Avg Approach Velocity (m/s)	ADA	3.73*	-0.065 (-0.12; -0.01)	-0.066 (-0.12; -0.01)
	505	4.32*	-0.038 (-0.08; 0.01)	-0.070 (-0.116; -0.02)
Avg Approach Momentum (kg*m/s)	ADA	7.55*	1.12 (-3.32; 5.56)	8.05 (3.60; 12.50)
	505	8.80*	3.81 (0.046; 7.58)	8.05 (4.29; 11.82)
Avg DEC (m/s^2^)	ADA	1.63	-0.036 (-0.20; 0.13)	-0.146 (-0.313; 0.02)
	505	8.64*	-0.14 (-0.253; -0.03)	-0.23 (-0.342; -0.12)
Avg Horizontal Braking Force (N)	ADA	0.27	3.62 (-9.58; 16.8)	-0.99 (-14.23; 12.3)
	505	0.89	-4.30 (-12.90; 4.27)	-5.58 (-14.2; 3.00)
Stopping Time (s)	ADA	0.59	0.04 (-0.03; 0.10)	0.01 (-0.06; 0.08)
	505	3.82*	-0.049 (-0.118; 0.02)	-0.098 (-0.167; -0.03)
Stopping Distance (m)	ADA	2.11	0.086; (-0.24; 0.41)	-0.234 (-0.556; 0.09)
	505	2.38	-0.08 (-0.35; 0.19)	-0.293 (-0.567; -0.02)
DEC COM Drop (cm)	ADA	5.93*	-1.05 (-1.94; -0.16)	-1.54 (-2.43; -0.65)
	505	1.39	-0.469 (-1.33; 0.39)	0.239 (-0.62; 1.10)
Avg Brake Step GCD (g)	ADA	35.8*	-0.950 (-1.31; -0.59)	-1.553 (-1.91; -1.19)
	505	27.6*	-0.929 (-1.28; -0.58)	-1.289 (-1.64; -0.94)
Avg Brake Step GCT (s)	ADA	2.48	-0.002 (-0.01; 0.01)	0.007 (-0.001; 0.02)
	505	2.35	0.007 (5.56e-5; 0.01)	9.30e-4 (-0.01; 0.01)
Avg Brake Step Hip Flexion at GC (deg)	ADA	1.17	-0.373 (-1.55; 0.81)	-0.914 (-2.10; 0.27)
	505	2.38	-0.837 (-1.78; 0.11)	-0.973 (-1.92; -0.03)
Avg Brake Step Knee Flexion at GC (deg)	ADA	2.40	-1.06 (-2.50; 0.38)	-1.59 (-3.03; -0.15)
	505	1.01	-0.725 (-2.16; 0.71)	-1.011 (-2.45; 0.42)
Avg Brake Step Position Relative to COM (cm)	ADA	2.86	-0.967 (-2.05; 0.12)	-1.279 (-2.37; -0.19)
	505	1.98	0.741 (-0.46; 1.94)	1.210 (0.01; 2.41)
Change of Direction Time (s)	505	2.40	0.005 (-0.02; 0.03)	0.025 (0.001; 0.05)
Plant Leg GCD (g)	505	1.79	0.369 (-0.109; 0.85)	-0.046 (-0.523; 0.43)
Plant Leg Hip Flexion Peak at GC (deg)	505	3.24*	-3.23 (-6.11; -0.34)	-3.29 (-6.18; -0.41)
Plant Leg Knee Flexion Peak at GC (deg)	505	0.61	-1.599 (-4.51; 1.31)	-0.500 (-3.41; 2.41)

Note: * = Statistically significant fixed effect omnibus test

DEC = Deceleration

ACC = Acceleration

COM = Center of mass

GC = Ground contact

Avg. = Average

deg = degrees

**Table 3:** Descriptive statistics for all metrics of interest across both assessments and all load conditions (Mean ± SE).

Metric	Test	CV%	0%	2% of BW	4% of BW
Avg Approach Velocity (m/s)	ADA	1.8	4.45 ± 0.06	4.39 ± 0.06	4.38 ± 0.06
	505	2.2	4.49 ± 0.07	4.45 ± 0.07	4.42 ± 0.07†^#^
Avg Approach Momentum (kg*m/s)	ADA	1.8	342 ± 11.7	343 ± 11.6	350 ± 11.6†‡^#^
	505	2.1	344 ± 11.8	348 ± 11.8	352 ± 11.8†^#^
Avg DEC (m/s^2^)	ADA	8.4	3.29 ± 0.09	3.23 ± 0.09	3.14 ± 0.09
	505	5.5	4.09 ± 0.07	3.95 ± 0.07†	3.86 ± 0.07†^#^
Avg Horizontal Braking Force (N)	ADA	8.4	250 ± 7.52	253 ± 7.37	249 ± 7.39
	505	5.4	313 ± 10.4	309 ± 10.3	308 ± 10.3
Stopping time (s)	ADA	8.7	1.20 ± 0.03	1.23 ± 0.03	1.21 ± 0.03
	505	11.1	1.39 ± 0.05	1.34 ± 0.05	1.29 ± 0.05†
Stopping distance (m)	ADA	13.7	4.14 ± 0.16	4.19 ± 0.15	3.90 ± 0.15
	505	9.3	5.33 ± 0.16	5.25 ± 0.16	5.03 ± 0.16
DEC COM Drop (cm)	ADA	12.2	12.6 ± 0.79	11.4 ± 0.78†	11.0 ± 0.79†^#^
	505	19.5	12.9 ± 1.28	12.4 ± 1.27	13.1 ± 1.27
Avg Brake Step GCD (g)	ADA	5.9	11.4 ± 0.48	10.5 ± 0.78†^#^	9.9 ± 0.48†‡^#^
	505	7.5	11.1 ± 0.35	10.2 ± 0.35†^#^	9.9 ± 0.35†^#^
Avg Brake Step GCT (s)	ADA	6.8	0.20 ± 0.01	0.201 ± 0.01	0.210 ± 0.01
	505	9.4	0.19 ± 0.01	0.19 ± 0.01	0.19 ± 0.01
Avg Brake Step Hip Flexion at GC (deg)	ADA	11.4	33.2 ± 2.30	32.8 ± 2.30	32.5 ± 2.30
	505	6.8	31.7 ± 2.61	30.7 ± 2.61	31.1 ± 2.61
Avg Brake Step Knee Flexion at GC (deg)	ADA	6.6	34.6 ± 1.49	33.6 ± 1.48	33.0 ± 1.48
	505	11.3	33.1 ± 1.43	32.3 ± 1.43	32.1 ± 1.43
Avg Brake Step Position Relative to COM (cm)	ADA	4.5	41.4 ± 0.99	40.3 ± 0.98	40.1 ± 0.98
	505	8.3	36.6 ± 1.29	37.3 ± 1.28	37.8 ± 1.28
Change of Direction Time (s)	505	4.4	1.38 ± 0.02	1.39 ± 0.02	1.41 ± 0.02
Plant Leg GCD (g)	505	15.9	6.79 ± 0.39	7.16 ± 0.38	6.74 ± 0.38
Plant Leg Hip Flexion Peak at GC (deg)	505	18.7	42.5 ± 3.23	39.3 ± 3.22	39.2 ± 3.21
Plant Leg Knee Flexion Peak at GC (deg)	505	12.5	62.4 ± 1.83	62.0 ± 1.80	62.7 ± 1.80

Note: † = significantly different from unloaded condition

‡ = significantly different from the 2% BW condition

# = %-difference that exceeds the CV% established in the 0% BW condition

DEC = Deceleration

ACC = Acceleration

COM = Center of mass

GC = Ground contact

Avg. = Average

deg = degrees

## Discussion

The aim of this study was to investigate the acute effects of lower limb wearable resistance on maximal horizontal deceleration biomechanics, across two different assessments. Participants performed ADA and 5-0-5 tests across three load conditions (unloaded, 2% of BW, 4% of BW), with load attached to the thighs and shanks. It was hypothesized that in the wearable resistance condition, the subject may express altered movement characteristics, and that while speculative, this could present as a potential training tool for sport science practitioners to use. Previous investigations across different athlete populations have documented the potential positive effects of training with lower body wearable resistance on running biomechanics [15, 21].

In line with our study hypothesis, significant between-load condition differences were found for respective metrics of interest, across both assessments. In the ADA test, during which athletes are instructed to maximally decelerate to a stop, following a maximal 10-meter sprint, participants exhibited significantly greater degrees of approach momentum in the 4% BW condition, compared to the unloaded condition, as well as the 2% BW condition. Fixed effect parameter estimates from the linear mixed effect model suggest an 8.05 kg*m/s increase in Avg Approach Momentum, when compared to the unloaded condition, in performing the ADA test with 4% BW on the lower body, as used in this study. Further, while not statistically significant, participants experienced decreases in Avg Approach Velocity. This finding seems to align with data from Feser et al. (2021) who proposed that athletes experienced small to moderate reductions in sprint velocities, and significant increases in force-velocity variables such as theoretical maximal horizontal force, when using thigh wearable resistance [15]. Harper et al. have defined horizontal deceleration performance as the athlete’s ability to reduce whole body momentum, within the constraints, and in accordance with the specific objectives of the task [2]. Therefore, while still speculative, increasing athletes approach momentum by loading the lower limbs with 4% of BW, may provide a stimulus, elevating the skill, and neuromuscular demand (i.e., braking force control, braking force attenuation) on the athlete, to efficiently reduce the increased whole-body momentum. From a statistical standpoint, participants did not exhibit changes in Avg DEC or Stopping Time, as well as Avg Horizontal Braking Force, which may be seen as positive, given the increased approach momentum. However, this finding might be explained by the slightly decreased approach velocity, since larger velocities have been suggested to present an opportunity to attain larger reductions in horizontal velocity [2, 38, 39]. Interestingly, in the unloaded condition, participants used a deceleration strategy consisting of a significantly lower COM drop, compared to the 4% BW condition. Previous research has highlighted that during maximal horizontal decelerations, maintaining a low COM, and anteriorly oriented foot placement to shift the base of support relative to the COM, in order to increase posterior braking impulse may have implications for reducing knee joint mechanical loading, and therefore could have implications for injury risk reduction [2, 25]. In line with the previous, in addition to a lower COM drop in the unloaded condition during the ADA test, participants also exhibited deceleration strategies in the unloaded condition that consisted of a more anteriorly oriented foot placement, as seen by an increased Avg Brake Step Position Relative to COM. This may suggest that in the unloaded condition, participants presented with a more optimal deceleration strategy in the ADA test. While not part of our primary aim, a supplementary analysis suggested a greater COM drop to be significantly related to faster COD times in the 5-0-5 tests. Lastly, and exhibiting the largest between-condition effect, participants showed significantly lower degrees of Avg Brake Step GCD in the 2% BW, and 4% BW condition, compared to the unloaded condition, as well as in the 2% BW condition compared to the 4% BW condition. This proposes that in the wearable resistance conditions, the participants lower limbs accelerated toward the ground at a lower magnitude, suggesting a more cautious ground-contact interactions. Interestingly, previous research on the acute effects of wearable resistance on locomotor tasks such as running has proposed significant increases in vertical ground reaction force magnitudes during sprint running with 5% BW or greater [18, 31]. Further, and more in line with our procedures, a recently published study investigated acute kinematic and kinetic changes to wearable resistance to COD performance in soccer players [22]. Similar to the previously mentioned studies, this study also suggested small increases in relative peak vertical propulsive ground reaction force, relative peak braking ground reaction forces relative peak total propulsive ground reaction force, and relative braking impulse [22]. However, what should be acknowledged, is that in this study, participants wore the additional load of 5% BW on the torso, instead of the lower body. While speculative, the findings of the present study may suggest that when additional load is attached to the lower limbs during maximal decelerative or COD tasks, athletes may exhibit different interactions with the ground, characterized by lower magnitudes of ground contact deceleration and therefore, potentially ground reaction force.

Similar to the ADA test, in the 5-0-5 tests, participants experienced significantly larger approach momenta in the 4% BW condition, compared to the unloaded condition. Additionally, participants presented with significantly lower Avg Approach Velocities in the 4% BW condition, compared to the unloaded condition. This finding aligns with previous research suggesting small to moderate reductions in sprint velocities, and increases in sprint times over 10-, and 50-meters, when using shank loading of 2% BW [15, 40]. Likely influenced by this reduction in Avg Approach Velocity, participants also exhibited significantly smaller Avg DEC magnitudes in the 4% BW condition, compared to the unloaded condition. Fixed effect parameter estimates suggest decreases of 0.23 m/s^2^ when athletes performed 5-0-5 trials in the 4% BW condition. Further, participants showed significantly lower degrees of GCD in the 4% BW condition, and 2% BW condition, compared to both the unloaded condition. Interestingly, this finding and the magnitude of difference between conditions tracks consistent across both assessments used in this study. From a statistical standpoint, metrics related to the participants deceleration technique (e.g., joint angles, vertical movements of the COM, brake step ground contact times, anterior placement of brake steps relative to COM) remained unchanged across conditions for the most part. While using a different COD angle and wearable resistance location, these findings align with previous reports, suggesting non-significant changes in movement technique when performing a COD maneuver with 5% BW attached the torso [22].

In an effort to explain the significantly decreased GCD’s during loaded running across both assessments, which do not align with previous findings suggesting increased ground reaction forces with wearable resistance [15, 30, 31], the authors speculate those to be associated with the underlying biomechanical differences between acceleration/sprint running, and horizontal decelerations. Compared to horizontal accelerations, during decelerative tasks, the athlete’s center of mass is positioned posteriorly, in relation to the point of contact. This is achieved by flexing at the hip, extending at the knee, and plantar flexing the ankle to generate a horizontal braking force upon ground contact, to effectively attenuate and disperse force across different joints in the body [4]. These decelerative brake steps therefore exhibit a distinct ground reaction force profile that is characterized by impact peak forces and loading rates that are in some cases up three times as high as during maximal horizontal accelerations, requiring athletes to possess sufficient neuromuscular qualities such as eccentric and reactive strength [5]. While speculative, and solely based on our data, when athletes are tasked with performing maximal horizontal decelerations in the 2% BW and 4% BW conditions, they exhibit a brake step profile characterized by lower ground contact decelerations. Furthermore, the primary muscles involved in horizontal decelerations are the quadriceps and gastrocnemius [40], both of which are muscles to which micro weights were superficially attached in our procedures. Couture et al. found increases in peak vertical ground reaction forces during treadmill running at 3.9 m/s, with loading of 5% BW or greater, however not with load attached to the lower body [31]. The same authors propose that a lower body load orientation potentially results in greater pre-activation/activation of the involved muscles which may lead to greater attenuation of impact forces and might help explain our depressed ground contact deceleration magnitudes in our loaded conditions [31]. Moreover, it may be thought of as a subconscious or intrinsic, protective mechanism induced by the added load to the lower limbs. However, further research is warranted investigating such hypotheses. It seems that the effects of wearable resistance on deceleration biomechanics are both site-specific (e.g., upper body vs lower body loading), as well as muscle action specific (e.g., braking vs. propulsion). To the authors knowledge, the only study reporting acute kinetic effects of wearable resistance loading (torso loading) on COD performance suggested small increases in ground reaction forces during the braking and propulsive phase of the plant step of a 45-degree cut, following a 5-meter acceleration [22]. Interestingly, in our study, findings suggested that while not reaching statistical significance, participants displayed greater magnitudes of plant leg GCD in the 2% BW condition, compared to the unloaded condition, while the unloaded condition and 4% BW condition were only marginally different from each other. This was likely influenced by the lesser knee flexion participants exhibited during the plant step in the 2% BW condition. The previous may suggest that the effects of wearable resistance on deceleration and COD biomechanics also differ between the preparatory steps in a COD maneuver, and the final foot contact, or plant foot action.

COD time, potentially being viewed as an outcome goal during the 5-0-5 was unaffected by the loading condition, supporting the notion that participants were able to maintain overall COD performance in the 5-0-5, while experiencing some changes in biomechanical characteristics. More specifically, in the 4% BW condition, participants exhibited significant reductions in Avg Approach Velocity, Avg Deceleration, Stopping Time, as well as Avg Brake Step GCD, while seeing significant increases in Avg Approach Momentum, while maintaining COD time. These findings give rise to additional research questions, investigating whether or not these acute lower body wearable resistance-induced biomechanical changes may provide a stimulus to improve horizontal deceleration and COD performance, when implemented as a training tool over extended periods of time.

While novel, this study was not without limitations, which should be acknowledged in interpreting the findings of the study. For one, this study consisted of recreationally trained athletes. Future investigations may aim to replicate findings with populations of higher trained athletes. Further, while our study displayed no significant interaction effect when gender was added to the model, future studies may aim for an even number of male and female participants or study a cohort of female athletes all together. Additionally, Avg Deceleration Phase GCD was quantified as the combination of IMU-derived acceleration values from the x, y, and z direction, making it difficult to further dissect deceleration strategies. Another limitation that should be considered is the lack of a direct measure of kinetic data. Future research may aim to quantify GCD in all three directions, respectively, while using inverse dynamics to estimate ground reaction forces, or through the use of ground-imbedded force platforms, making for a direct measure. Furthermore, future studies may aim to explore further loading conditions, as well as the acute effects of different loading locations (e.g., thigh vs. shank placement). Lastly, in order to suggest the use of lower limb wearable resistance as a potential training tool for developing maximal horizontal deceleration as well as COD performance, longitudinal data is required on athlete populations using this specific modality.

## Conclusion

In summary, the findings presented in this study suggest that acutely, the addition of load to the anterior and posterior thigh and shank of the participants induced significant biomechanical changes in both the ADA test, and 5-0-5 test. In the ADA test, in the 4% BW condition, participants exhibited significantly greater degrees of Avg Approach Momentum, as well as significant reductions in deceleration phase COM drop, and Avg Brake Step GCD in both the 2% BW, and 4% BW condition, compared to the unloaded condition. In the 5-0-5 test, participants experienced significant reductions in Avg Approach Velocity, Avg DEC, and Stopping Time in the 4% BW condition compared to the unloaded condition. Similar to the ADA test, participants also experienced significant reductions in Avg Brake Step GCD in both the 2% BW and 4% BW conditions, and significant increases in Avg Approach Momentum in the 4% BW condition, compared to the unloaded condition. In the 5-0-5 tests, it seems that participants were able to maintain movement characteristics, and overall performance (COD time), while experiencing changes in other biomechanical parameters. Future investigations are warranted to further explore if the use of lower limb wearable resistance could present as an effective training tool in enhancing athlete’s horizontal deceleration and COD performance.
